# Automated Indexing of Internet Stories for Health Behavior Change: Weight Loss Attitude Pilot Study

**DOI:** 10.2196/jmir.3702

**Published:** 2014-12-09

**Authors:** Ramesh Manuvinakurike, Wayne F Velicer, Timothy W Bickmore

**Affiliations:** ^1^College of Computer and Information ScienceNortheastern UniversityBoston, MAUnited States; ^2^Cancer Prevention Research InstituteUniversity of Rhode IslandKingston, RIUnited States

**Keywords:** behavioral medicine, natural language processing, animation, consumer health, health informatics, self-efficacy

## Abstract

**Background:**

Automated health behavior change interventions show promise, but suffer from high attrition and disuse. The Internet abounds with thousands of personal narrative accounts of health behavior change that could not only provide useful information and motivation for others who are also trying to change, but an endless source of novel, entertaining stories that may keep participants more engaged than messages authored by interventionists.

**Objective:**

Given a collection of relevant personal health behavior change stories gathered from the Internet, the aim of this study was to develop and evaluate an automated indexing algorithm that could select the best possible story to provide to a user to have the greatest possible impact on their attitudes toward changing a targeted health behavior, in this case weight loss.

**Methods:**

An indexing algorithm was developed using features informed by theories from behavioral medicine together with text classification and machine learning techniques. The algorithm was trained using a crowdsourced dataset, then evaluated in a 2×2 between-subjects randomized pilot study. One factor compared the effects of participants reading 2 indexed stories vs 2 randomly selected stories, whereas the second factor compared the medium used to tell the stories: text or animated conversational agent. Outcome measures included changes in self-efficacy and decisional balance for weight loss before and after the stories were read.

**Results:**

Participants were recruited from a crowdsourcing website (N=103; 53.4%, 55/103 female; mean age 35, SD 10.8 years; 65.0%, 67/103 precontemplation; 19.4%, 20/103 contemplation for weight loss). Participants who read indexed stories exhibited a significantly greater increase in self-efficacy for weight loss compared to the control group (*F*_1,107_=5.5, *P*=.02). There were no significant effects of indexing on change in decisional balance (*F*_1,97_=0.05, *P*=.83) and no significant effects of medium on change in self-efficacy (*F*_1,107_=0.04, *P*=.84) or decisional balance (*F*_1,97_=0.78, *P*=.38).

**Conclusions:**

Personal stories of health behavior change can be harvested from the Internet and used directly and automatically in interventions to affect participant attitudes, such as self-efficacy for changing behavior. Such approaches have the potential to provide highly tailored interventions that maximize engagement and retention with minimal intervention development effort.

## Introduction

### Background

The importance of health behavior change on public health has been well established, with lifestyle behaviors such as smoking, physical activity, and diet widely acknowledged to have major impacts on morbidity and mortality [1]. Automated health behavior change interventions have the promise of providing much greater reach and lower cost compared with interventions delivered by professionals. However, automated interventions—whether delivered through the Web, mobile devices, telephone via interactive voice response (IVR), or other media—can be costly and time-consuming to author or adapt [2]. In addition, tailoring intervention messages to user characteristics, such as age, race, gender, geographic locale, literacy level, self-efficacy, and stage of change, is known to be important and lead to more efficacious outcomes [3]. However, manually authoring messages for every combination of tailoring parameters makes development even more expensive and unwieldy despite the existence of automated tools to assist in the process [2].

In this paper, we explore a fundamentally new approach to developing automated health behavior change interventions by leveraging existing health message content on the Internet. Specifically, we aim to repurpose existing personal stories of health behavior change reported in blogs, bulletin board posts, and publicly available emails as the source messages for automated health behavior change systems. For blogs alone, more than a million posts are made each day on the Web, with the most common topic being stories of personal experience [4].

Personal stories of health behavior change represent a particularly powerful messaging mechanism. Heath information that is grounded in the personal experience of members of a community is more likely to be engaging and less likely to be dismissed [5]. Curated, edited, and packaged personal stories have also been used in several health behavior interventions. For example, Houston et al [6] videotaped stories told by individuals with hypertension and used these in a DVD-based intervention to help other patients with hypertension control their chronic condition. In a randomized clinical trial with 299 participants, those receiving the story-based intervention had significantly lower blood pressure after 3 months compared to a control group that received attention-control DVDs [6]. In addition, many individuals prefer getting certain types of health information via informal communication with others rather than from health professionals. A recent poll by the Pew Internet and American Life project reported that 43% of respondents preferred practical advice about day-to-day health situations from medical professionals, whereas a surprising 46% preferred to get this advice from other informal sources and sources such as family, friends, and fellow patients [7].

There are 3 primary challenges in our approach: (1) determining how to find and curate stories that provide helpful information or motivation (ie, filter out misinformation and stories that could potentially be counterproductive or even do harm), (2) determining how to select the best story to provide to a user at a given time (the “indexing” problem), and (3) determining the best medium and manner of telling a selected story to a user. Our central focus in this paper is on the second problem, but we also explore 2 different mediums in our summative evaluation to provide some preliminary information to address the third problem. Succinctly stated, the indexing problem is how to choose the best story from among potentially millions of possibilities to tell a given user at a given time to maximize their likelihood of achieving a specified health behavior criterion at some time in the future.

We focused our pilot work in this area on weight loss promotion. Overweight and obesity have reached epidemic proportions in the United States, with 68.5% of adults overweight or obese (body mass index [BMI] 25 or greater), 34.9% obese (BMI ≥30), and 6.5% extremely obese (BMI ≥40) [8]. These conditions have been implicated in a range of health conditions, including heart disease, stroke, type 2 diabetes mellitus, and some types of cancer [9], and the economic impact of obesity alone was estimated at US $99.2 billion in 1995 [10].

In the remainder of this paper, we describe the development and validation of a health behavior change story-indexing algorithm informed by theories of health behavior change and health communication. We then describe an evaluation study in which we assess the impact of automatically indexed personal stories of weight loss on psychological constructs from behavioral medicine; specifically, stage of change, self-efficacy, and decisional balance for weight loss.

### Related Work in Story Indexing

Story indexing has its roots in early story understanding systems in which story indexes were hand-coded for intelligent retrieval and inference [11]. Early work on story understanding focused on information extraction to identify the stories that contained specific topics of interest [12]. More recently, researchers have employed statistical methods of text classification to collect and classify stories from large online datasets. For example, Gordon et al [4] created a classifier that was able to identify a million personal weblog entries from the Spinn3r dataset. Some researchers also explored automated story indexing for advice giving. Domeshek et al [13] developed a system that used 500 indexes to enable retrieval of stories to be used for social advice.

### Related Work in Storytelling by Conversational Agents

Several prior studies have now demonstrated the efficacy of health interventions delivered by automated natural language dialog [14]. In particular, animated conversational agents that simulate face-to-face counseling sessions with a health professional have been shown to be well-accepted and a good vehicle for conveying the social dimensions of health messages [15,16], and have also been used to deliver stories to users in health care interventions [17,18].

### Story Corpus

We collected 260 stories from websites including PatientsLikeMe [19], Experience Project [20], HealthTalkOnline [21], and About.com [22], where people frequently write about their experiences with health behavior change. Table 1 shows the places from which the stories were sourced. The stories were hand-selected (by the first author) to be first-person accounts of successful attempts to work toward weight loss behavior change, evidenced by self-reported improvements in motivation, intention to change, or specific behaviors.

The stories did not go through any other filtering process other than the manual selection. The stories were relatively short, with a mean 327 (SD 211, range 44-1076) words. An excerpt from a story in the corpus:

My name is XXXX and I am 32 years old. Worst moment was seeing my son, pick up my bad eating habits. As a 1-year-old, he was having fries at McDonald's because that's what he saw me putting in my mouth. I read an article in my husband's health magazine about BMI and life expectancy; it said that being obese can shorten your life by 5 to 10 years. That was scary. I didn't want to miss out on any moments with my son just because of poor eating patterns. After that, every time I was tempted to have a fry, I'd think, “I could be here a little bit longer if I just eat better”...Weigh yourself twice a day every morning and night. It's the best way to stay on track. Exercise, even if it's only for 10 minutes. Anything is better than nothing. I also lift weights to boost my metabolism.

**Table 1:** Sources of the stories and the number of stories selected from the sources.

Website (sources of stories)	Number of stories
Experience Project [20]	40
PatientsLikeMe [19]	40
HealthTalkOnline [21]	40
About.com [22]	23
Weight Loss Resources [23]	20
Yahoo! Lifestyle [24]	20
Blubberbusters [25]	20
3 Fat Chicks on a Diet! [26]	15
Weight Loss Success Stories [27]	12
Fitbie [28]	9
People [29]	8
Good Housekeeping [30]	5
Ladies’ Home Journal [31]	5
Woman’s Day [31]	3
Total	260

### Story Indexing

Our indexing algorithm was designed to select the best story to tell a particular user at a particular time based on theories from behavioral medicine, health communication, and linguistics. From behavioral medicine, we included indexes based on information from the transtheoretical model (TTM; also known as the “stages of change” model) to select stories that help advance a user in their behavior change trajectory. From health communication, we included indexes based on tailoring theory to select stories that were as relevant as possible to the user’s characteristics. From linguistics, we included indexes that selected stories based on assessments of story quality and positive affective tone. We describe each of these indexing features subsequently.

### Indexing Features from the Transtheoretical Model of Health Behavior Change

The TTM of health behavior change posits that individuals change by progressing through a series of 5 well-defined stages, and that the messages and actions that are the most helpful to an individual at a given time are based on the stage they are in [32]. The 5 stages of change are precontemplation (people are not intending to take action in the foreseeable future), contemplation (people are intending to change soon), preparation (people are intending to take action in the immediate future), action (stage in which people have made specific modifications in their lifestyles), and maintenance (people are working to prevent relapse).

**Figure 1:**
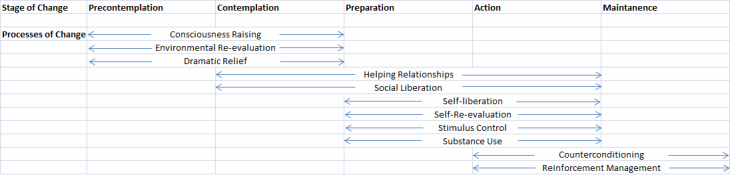
Relation between stages and processes of change for weight loss [32].

The TTM has been applied to a wide range of health behaviors, including weight loss [33]. In addition to the 5 stages, TTM defines 12 categories of specific behavior change activities (“processes of change”) and posits that the processes that are most helpful to a given person at a given time depend on the individual’s stage and the behavior being changed (Table 2). For example, consciousness raising (eg, researching the benefits of weight loss) is most helpful for individuals who are not yet considering change (precontemplation stage), whereas stimulus control (eg, putting a gym bag near the door as a reminder to exercise) is most helpful for those actively taking steps to change (action stage). Similarly, dramatic relief (eg, emotionally charged portrayals of the problems of overweight people) and environmental re-evaluation (eg, reflecting on the impact the behavior has on the environment, such as society’s food supply) are most useful for precontemplators (Figure 1 shows the relationships for weight loss).

The TTM also posits that self-efficacy (the degree of confidence an individual has to change their behavior, based on social cognitive theory [34]) and decisional balance (the perceived pros and cons of change) [35] covary in systematic ways with stage of change, but with the exact nature of the relationship depending on the particular behavior being changed. In general, self-efficacy and pros increase monotonically, whereas cons decrease monotonically as an individual progresses through the 5 stages of change.

The stage of change construct reflects information about an individual’s intention to change their behavior in the future (precontemplation, contemplation, preparation) or the duration for which they have maintained change at a criterion level (action, maintenance). This specific temporal information rarely occurs in anecdotal stories of change and, even if it is explicitly or implicitly related in a story, it would require very sophisticated natural language processing to accurately extract. In comparison, specific behavior change activities (processes) are frequently mentioned in stories and are significantly easier to identify compared to information explicitly relating the author’s stage of change. Thus, in our approach we use text classification methods to automatically identify processes of change in stories as features for indexing. When the system indexes the best story to tell for a given user, it uses information about the user’s stage of change and the most relevant processes for that stage to index the best stories to tell.

**Table 2:** Processes of change used in indexing algorithm [32].

Processes of change	Description
1. Consciousness raising	Attempt to seek out information concerning their problem behavior
2. Dramatic relief	Increased emotional experiences followed by reduced affect if appropriate action can be taken
3. Substance use	Use of medication
4. Social liberation	Increase in social opportunities
5. Self–re-evaluation	Cognitive and affective assessments of one’s self-image
6. Stimulus control	Removes cues for unhealthy habits and adds prompts for healthier alternatives
7. Helping relationship	Combine caring, trust, openness, and acceptance as well as support for the healthy behavior change
8. Counter conditioning	Learning of healthier behaviors that can substitute for problem behaviors
9. Reinforcement management	Consequences for taking steps in a particular direction
10. Self-liberation	Belief that one can change and the commitment and recommitment to act on that belief
11. Environmental re-evaluation	Affective and cognitive assessments of how the presence or absence of a personal habit affects one’s social environment

### Indexing Features From Tailoring Theory

Tailored health messages are those that are uniquely individualized to each person [36]. The most common approaches to tailoring involve selecting or adapting messages based on user characteristics, such as age, gender, race, and ethnicity. Message tailoring can be readily automated and computer-based tailored message interventions have been developed and evaluated. Computer-tailored messages have been shown to be significantly more effective at changing attitudes and behavior compared to nontailored messages in a number of studies [3]. Tailoring is thought to work by making messages more subjectively relevant and thus increasing the likelihood that individuals will process and internalize them [36].

In our approach, we used text classification methods to automatically identify demographic characteristics of authors based on the text of their stories, including age, gender, education level, and race. When the system indexed the best story to tell for a given user, it used information about the user’s demographics to index the best stories to tell by preferring stories whose authors matched the user along these dimensions.

### Affective Tone Indexing Feature

Stories that have an overall positive emotional tone are known to be more engaging and impactful than stories that are negative [37]; in addition, emotion can play a significant role in certain processes of change (eg, dramatic relief). Thus, we used text classification techniques to automatically compute the overall emotional tone (“sentiment”) of a story, both as a process-specific feature and as a user-invariant quality metric to prefer stories that are positive.

### Text Coherence Indexing Feature

Finally, we used an existing algorithm (Coh-Metrix) to automatically compute the coherence of a given story as a rough measure of writing quality [38]. The Coh-Metrix scores were calculated for stories using the online Coh-Metrix tool. This metric indicates how semantically meaningful, continuous, and understandable a story is and, like affective tone, is used as a user-invariant quality metric to prefer stories that are more coherent. Stories with higher Coh-Metrix scores were selected above the stories with lower scores only when the stories scored equally on all other indexes.

### Indexing Algorithm

A given story may describe several behavior change activities that span more than 1 process of change; thus, it may be important to identify all processes of change mentioned in a story. Use of a multiclass classifier that combines information from 12 independent binary classifiers may seem to be a reasonable approach. However, analysis of the set of processes of change that are most useful for each stage of change for weight loss yields only 5 unique sets of processes that need to be identified, decreasing the complexity of the text classification problem from 12 classes to 5 (Table 3). In sum, we have developed a relevant stage of change classifier using the processes of change as features.

**Table 3:** Relevant stage of change classifier [19].

Class	Processes of change	Stage of change	Classification accuracy	F-measure
1	Consciousness raising, environmental re-evaluation, dramatic relief	Precontemplation	0.88	0.91
2	Consciousness raising, environmental re-evaluation, dramatic relief, helping relationship, social liberation	Contemplation	0.94	0.96
3	Helping relationship, social liberation	Preparation	0.87	0.93
4	Helping relationship, social liberation, self-liberation, self–re-evaluation, stimulus control, substance use, counter conditioning, reinforcement management	Action	0.98	0.95
5	Counter conditioning, reinforcement management	Maintenance	0.84	0.91

### Indexing Algorithm Training and Accuracy Results

The stage of change classification algorithm training was performed using Adaptive Boosting (AdaBoost) [39] with weak learners using Weka [40]. Support vector machine, Naive Bayes, and regression classifiers were tried before settling with AdaBoost. The classifier producing the best accuracy without overfitting was selected for the task. The set of features used for classification included the presence of keywords for each process of change, sentiment scores of stories, part of speech (POS) tags, the position of keywords within a story, and any categorization tags associated with the story. Apache OpenNLP was used to obtain the parts of speech (eg, noun, verb) from the stories. The POS bigram and unigram counts in the story formed a set of features extracted. The keyword sets for each of the processes of change were formed from the stories. This feature was a binary valued indicator function. The value indicated if the story contained at least 1 keyword present in the keywords set defined for each process of change. As an example, the keywords for the substance use process of change (use of medication) included drugs, pills, medicine, tablets, etc. The indicator feature was generated to indicate the presence of substance use feature in the story. Similarly, the keywords for counterconditioning (learning of healthier behaviors that can substitute for problem behaviors) contained alter, change, substitute, lieu, etc. Helping relationship (combining caring, trust, openness, and acceptance as well as support for the healthy behavior change) keywords included mother, doctor, nurse, friend, help, motivate, etc. These indicator functions for each of the processes of change were constructed .and feature values were generated which indicated the presence of process of change keywords in the story. For each of the keyword lists, the position feature was constructed which indicated the part of the story (beginning, middle, or end). This function used 3 values which indicated if the keywords were present in the first, second, or the last third of the story based on the word count. For instance, if the keywords for a particular process of change was present in the first 100 words of a 300-word story, the feature took the value of “beginning.” The same feature took the value of “end” if the substance use keyword was present in the last 100 words of the story. Stories containing dramatic relief processes of change generally contained higher sentiment scores. Numbers of noun and verb phrases were also used as a feature set obtained from Apache OpenNLP.

We trained our text classifiers on the corpus of 260 stories using calibration input from a crowdsourcing website. The calibration study was conducted using 260 participants on Amazon’s Mechanical Turk. Northeastern University’s institutional review board (IRB) approved the study. Participants each read a story from the corpus and answered 44 yes/no questions about it based on an existing processes of change questionnaire for weight loss [41]. These questions helped identify the processes of change from the stories by mapping each response to separate processes of change. The responses to these questionnaires were mapped to the individual processes of change of TTM. The interrater reliability test was conducted on the mapped processes of change rather than the responses to the 44 questions. We conducted interrater reliability tests for the processes of change on 10 randomly selected stories by having 2 separate groups of users provide feedback. The resulting kappa was 0.80, indicating adequate reliability.

We used 10-fold cross-validation methodology to assess match accuracy on the story corpus. These values ranged from 83.8% to 98.1% for the 5 categories (Table 3).

We also constructed and trained text classifiers to automatically classify the gender of the story author using the same methodology as described previously. The age and race of the author were classified using regular expressions and their education level was computed using the story’s readability score [38].

The final story-indexing algorithm used a user’s demographic information (age, gender, education level, and race) and stage of change for weight loss (from a short questionnaire [35]) and scored each story in the corpus indicating the relative degree of relevance of the story to the user. The score was a weighted sum comprised of terms for degree of match between the user’s demographic information and stage of change and the corresponding feature automatically computed from the story, as well as terms for the overall positive affect (sentiment) and quality (coherence) of the story. We weighted stage of change 5 times greater than the other terms in the score calculation.

## Methods

### Summary

We conducted an evaluation study to determine the impact of indexed stories on the health behavior change attitudes of individuals. This study was also conducted using a crowdsourcing site and was approved by Northeastern University’s IRB. In this study, we sought to compare the impact of stories retrieved from the corpus by the indexing algorithm described previously and compared them to the impact of stories selected at random. We also sought to evaluate 2 different modalities for delivering the stories to individuals based on our prior work in using conversational agents for storytelling: an animated conversational agent vs text. Thus, our hypotheses were that for individuals interested in losing weight:

1. Indexed stories will have significantly greater impact than randomly selected stories on (1) changes in self-efficacy and decisional balance for weight loss and (2) ratings of story understandability, enjoyment, and identification with the story author.
2. Stories told by an animated conversational agent will have significantly greater impact than stories displayed in text on (1) changes in self-efficacy and decisional balance for weight loss and (2) ratings of story understandability, enjoyment, and identification with the story author.

To evaluate these hypotheses, we conducted a 2×2 full-factorial between-subjects experiment in which participants were randomized into 1 of 4 conditions according to 2 factors: (1) selection—stories were either selected using the indexing algorithm described previously (indexed) or selected at random (random) and (2) medium—stories were delivered either by a conversational agent (agent) or stories delivered in text (text).

### Recruitment

The evaluation study was conducted using participants on Amazon’s Mechanical Turk. Participants were required to be US-based “Master Turkers” (most experienced) and willing to lose weight.

### Measures

The following self-report measures were assessed at enrollment and at the end of the intervention:

1. Stage of change for weight loss was assessed at enrollment and at the end of the intervention using the weight loss stages of change short form [33,35].
2. Weight loss self-efficacy was measured at enrollment and at the end of the intervention using a validated 10-item questionnaire [42].
3. Weight loss decisional balance was measured using a 20-item questionnaire [35].

In addition, the following measures were assessed at the end of the intervention:

1. Enjoyment of stories was measured using a single-item scale question (“How enjoyable was the story?”).
2. Understandability of stories was assessed using items from a technology acceptance scale [43].

Identification with story was measured using 2 single-item scale questions (“How much can you identify with the story author?” and “How close were the experiences of the person in the story with yourself?”).

### Protocol

After passing our eligibility criteria and agreeing to an unsigned informed consent, participants filled out pretest questionnaires measuring demographics, stage of change, self-efficacy, and decisional balance.

**Figure 2:**
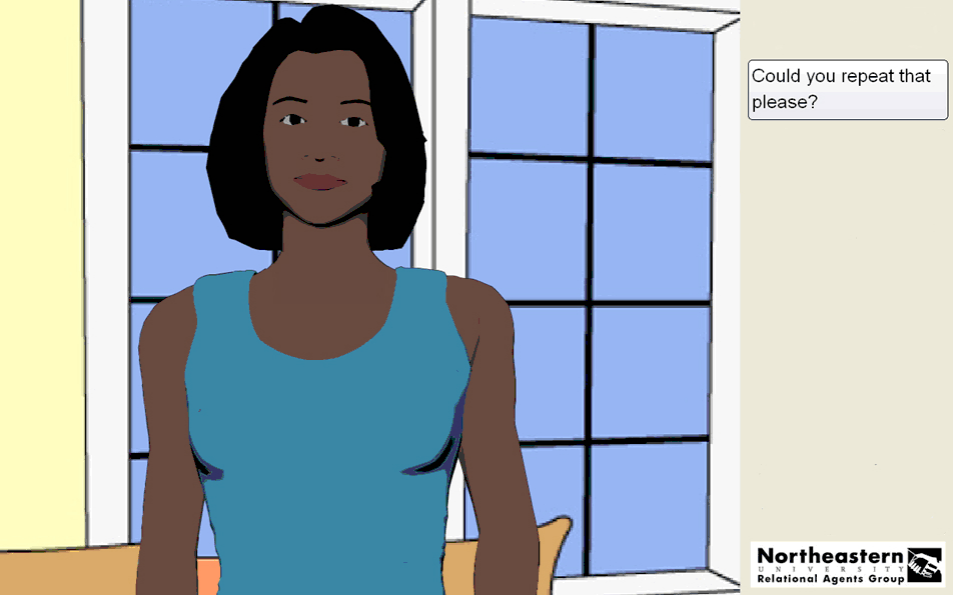
Animated conversational agent interface.

Participants were then presented with the first story, either selected using the indexing algorithm (for indexed condition) or at random (for random condition). In text conditions, the story was simply displayed in a large text box. In agent conditions, an animated conversational agent was displayed that told the story verbally using synthetic speech and synchronized nonverbal conversational behavior (Figure 2) [44]. Participants were then presented with a questionnaire assessing enjoyment, understandability, and identification with the characters mentioned in the story.

Participants were then presented with a second story (in the same study treatment as the first) followed by a second administration of the questionnaire assessing enjoyment, understandability, and identification. Finally, participants were given a second administration of the self-efficacy and decisional balance questionnaires.

### Statistical Analysis

Data were analyzed using 2×2 ANOVAs in SPSS (IBM Corp, Armonk, NY, USA) with selection and medium as the independent factors.

## Results

### Participants

Participant demographics are shown in Table 4. The 107 participants were recruited from 29 states in the United States. Only 4 of 107 participants withdrew from the study, all in the agent condition (2 in indexed, 2 in random). Therefore, 103 participants completed the entire study and were included in the final analysis; 53.4% (55/103) were female, the mean age was 34.85 (SD 10.8) years, and most were well educated (all had at least a high school education and 13.6%, 14/103 had graduate degrees).

**Table 4:** Participant demographics.

Measure		All participants	Agent		Text	
		(N=103)	Indexed (n=35)	Random (n=19)	Indexed (n=26)	Random (n=23)
Sex (female), n (%)		55 (53.4)	18 (51)	10 (53)	15 (58)	12 (52)
Age (years), mean (SD)		35.2 (11.1)	36.1 (12)	33.5 (11)	36.9 (10)	33.3 (11)
Race (white), n (%)		85 (82.5)	31 (89)	15 (79)	20 (77)	17 (74)
**Education, n (%)**						
	<High school	0 (0.0)	0 (0)	0 (0)	0 (0)	0 (0)
	High school	18 (17.5)	7 (20)	3 (16)	3 (12)	5 (22)
	>High school	85 (82.5)	28 (80)	16 (84)	23 (89)	18 (78)
**Stage of change, n (%)**						
	Precontemplation	67 (65.0)	24 (69)	12 (63)	17 (65)	14 (61)
	Contemplation	20 (19.4)	5 (14)	5 (26)	5 (19)	5 (22)
	Preparation	12 (11.7)	4 (11)	2 (11)	3 (12)	3 (13)
	Action	4 (3.9)	2 (6)	0 (0)	1 (4)	1 (4)

### Evaluation Outcomes

Table 5 summarizes results from the study. There were no significant effects of study manipulations on change in decisional balance for weight loss. There was a main effect of selection on change in self-efficacy for weight loss (*F*_1,107_=5.5, *P*=.02) with indexed stories leading to significantly greater increases in self-efficacy compared to random stories. There was no effect of medium on change in self-efficacy (*F*_1,107_=0.04, *P*=.84) and no significant interactions.

There was a significant main effect of medium on enjoyment of stories (*F*_1,98_=22.3, *P*<.001), such that participants enjoyed reading stories themselves (text condition) significantly more compared to stories read by the agent (agent condition). There was no significant effect of selection on enjoyment.

There was a significant main effect of medium on identification with the story (*F*_1,98_=47.2, *P*<.001), such that participants identified significantly more with stories in the text condition compared to those in the agent condition. There was also a trending main effect of selection on identification (*F*_1,98_=3.0, *P*=.09) with participants identifying more with indexed stories compared to those selected at random.

Finally, there was a significant interaction of selection and medium on the rated understandability of stories (*F*_1,98_=4.5, *P*=.04), such that participants receiving indexed stories rated those read by the agent as more understandable, whereas those who received random stories rated those delivered in text as more understandable. However, there was also a strong main effect of medium on understandability (*F*_1,98_=82.8, *P*<.001) with participants in the agent group rating the understandability of their stories more highly overall compared to those in the text group.

**Table 5:** Primary outcomes by study condition.

Measure	Agent, mean (SD)		Text, mean (SD)	
	Indexed	Random	Indexed	Random
Self-efficacy (post-pre)	5.54 (4.54)	0.68 (7.40)	3.54 (3.66)	0.87 (3.98)
Decisional balance (post-pre)	0.37 (6.19)	1.89 (9.04)	0.27 (5.61)	–0.35 (2.23)
Enjoyment	2.15 (0.88)	2.45 (1.05)	3.15 (0.83)	2.89 (0.64)
Understandability	3.13 (0.85)	2.82 (0.99)	1.31 (0.69)	1.87 (0.64)
Identification	1.58 (0.73)	1.55 (0.71)	2.89 (0.86)	2.32 (0.63)

## Discussion

### Principal Results

Our first hypothesis (indexed stories will have significantly greater impact than randomly selected stories) received partial support, with indexed stories leading to significantly greater increases in self-efficacy and higher ratings of story understandability compared to stories selected at random. There was also a trend for indexed stories to lead to greater identification ratings. The lack of significant change in decisional balance could either be due to the complex relationship between self-efficacy and decisional balance (participants may not have been at the appropriate stage of change to see movement in both self-efficacy and decisional balance) or due to indexed stories acting uniquely on self-efficacy (confidence) without impacting decisional balance (perceived advantages or disadvantages of change).

However, our second hypothesis (stories told by an animated conversational agent will have significantly greater impact than stories displayed in text) received mixed support in the experiment. Although participants rated the understandability of stories more highly when read by an agent, they enjoyed them more and identified with them more when they read the stories from text. Medium had no effect on health behavior change attitudes.

### Limitations

Relative to the millions of stories available on the Internet, the size of our experimental corpus of stories was tiny. However, we were able to demonstrate significant changes in health attitudes just with this small collection.

Our pool of participants was also very small and highly biased demographically. Although both genders were represented equally, Turkers are known to be younger, better educated, and earn more than the general US population [45].

### Comparison With Prior Work

Houston et al [6] evaluated the impact of manually curated, edited, and packaged personal stories on health behavior change and demonstrated significant impacts on hypertension control compared to controls. However, this work is representative of the majority of research in behavioral medicine in which months or years are spent by experts developing and refining an intervention. In our approach, much of the intervention design is automated.

Lu [46] conducted a study in which she evaluated the effects of simulated blog posts on the health attitudes and intentions of individuals who read them. The posts were handcrafted to be either narrative (story) or nonnarrative, and to match study participants along various dimensions (“source similarity” which we have referred to as “tailoring”). Lu found that the tailoring effect was stronger in nonnarrative than narrative blogs. When the blogs were nonnarrative, those with health-related similarities were more effective at changing health-related intentions than those with non-health-related similarities [46]. Although this study provides useful information that may improve our story-indexing approach, its objectives are fundamentally different from our goal of automatically selecting the best story to tell a given user from a collection of existing stories on the Internet.

### Conclusions

We demonstrated the feasibility of automatically indexing stories of health behavior change gathered from the Internet and its ability to positively impact the health attitudes of individuals who read the stories.

We plan to continue improving our indexing algorithm with better features, improved machine learning methods, and a much larger training corpus. We also plan to investigate automating the process of identifying health behavior change stories on the Internet based on existing work on identifying personal stories in blog posts [4]. Automatically identifying and selecting stories that meet a particular individual’s current needs and doing this in the context of a longitudinal health behavior change intervention represents a rich area of future research.

## Abbreviations

BMI: body mass index
IVR: interactive voice response
POS: part of speech
TTM: transtheoretical model
